# Impact of Rodenticides on the Coagulation Properties of Milk

**DOI:** 10.3390/foods7040057

**Published:** 2018-04-07

**Authors:** Salam A. Ibrahim, Tom Tse

**Affiliations:** Food Microbiology and Biotechnology Laboratory, Food and Nutritional Sciences Program, North Carolina Agricultural and Technical State University, Greensboro, NC 27411-1064, USA; tsesiufai@hotmail.com

**Keywords:** rat poison, milk contamination, milk coagulation, rennet coagulation time, rodenticides

## Abstract

In this study, we investigated the impact of the rodenticides (strychnine, bromadiolone, and brodifacoum) on milk pH, rennet coagulation time (RCT), and coagulum strength. Sub-lethal amounts of strychnine and bromadiolone produced an unnaturally large change in milk pH, compared to brodifacoum and brodifacoum on milk coagulation properties. All three studied rodenticides significantly affected RCT and coagulum strength. The presence of sub-lethal amounts of each individual rodenticide increased RCT by an overall mean of 17% (*p* < 0.001). Rodenticide contamination decreased coagulum strength by an overall mean of 26% (*p* < 0.05). Our results suggest that such changes could be noticeable at the farm, thus, potentially averting the mixture of contaminated milk with the tanker supply, and preventing downstream distribution to consumers.

## 1. Introduction

Dairy products are a significant part of typical U.S. diets [1]. In particular, milk’s broad reach, in addition to its multi-stage processing chain, makes it especially vulnerable to intentional contamination. The processing of cheese begins with the coagulation of milk [2]. The coagulated milk is then further processed into the desired cheese product.

The coagulation of milk is a multi-stage process of overlapping physico-chemical changes. Cheese-making often begins with the aspartyl protease chymosin (also called rennet). Chymosin hydrolyzes the amide bond between Phe105 and Met106 of the κ-casein milk protein [3]. The catalyzed hydrolysis reaction releases the hydrophilic tail of κ-casein and the hydrophobic effect then drives aggregation of the casein micelles [2].

Milk coagulation depends on several parameters, including pH and temperature. The normal pH range of milk is between 6.4 and 6.8 [4]. A decrease in pH accelerates milk coagulation. At low pH, protonation of casein phosphate groups becomes more favorable, reducing zeta potential and, thus, accelerating coagulation [5]. However, a robust buffer prevents drastic pH changes in milk [6].

Overall, enzymatic coagulation is a physically apparent transformation driven by an underlying chemical one. The simultaneous cascade of events yields multiple points at which contaminant may play a role, whether in the pH, enzymatic hydrolysis, or hydrophobic assembly. In addition, more than a dozen antibiotic residues were found to significantly impact the milk coagulation process [7]. Thus, in this study, we hypothesized that rodenticides could contaminate the milk on farms and can affect the milk coagulation. During the coagulation process, several parameters are monitored for quality control purposes, including milk pH, gel strength, and rennet coagulation time (RCT) [8]. Of course, these parameters vary from day to day due to natural variations in milk quality and among individual cows [9]. However, changes that far exceed those expected due to normal variation may serve as preliminary detectors for the presence of milk contaminants. Since these properties are already monitored, using them to detect rodenticides would not require expensive retrofitting of existing cheese manufacturing technology.

Previous investigations have considered the impact of medicinal drug residues on coagulation properties of milk [7], finding that coagulation time and gel firmness can be significantly affected by chemical contamination. Antibiotic residues in milk have also been detected and quantified with high performance liquid chromatography (HPLC) [10]. Several on-line techniques are available for detecting antibiotics in milk, including liquid chromatography [11]. In addition, past studies have found that HPLC can identify minute quantities of strychnine in milk [12,13]. Toxic combustion by-products, such as polycyclic aromatic hydrocarbons, have also been detected in milk by using mass spectrometry and gas chromatography [14]. More sophisticated techniques involving gold nanoparticles can detect the presence of other toxic substances, such as melamine [15]. However, these techniques are costly and cannot be implemented until potentially contaminated milk has already arrived at the dairy processing plant.

Thus, we investigated a preliminary detection method for an earlier point in the supply chain: the farm itself. Coagulation parameters can be determined with a small sample from the farm’s bulk storage tank, flagging potentially contaminated samples for isolated analysis. Such an early intervention could prevent further processing into dangerous cheese on the farmstead or the introduction of contaminated milk into the tanker truck. We examined three potent rodenticides for their impact on milk coagulation: strychnine, bromadiolone, and brodifacoum. Although strychnine is a highly bitter substance, unwitting ingestion has been documented [16,17]. Notably, these rodenticides are widely used in agriculture and are accessible on farmsteads [18]. High proximity, availability, and an inconspicuous appearance as a white powder heighten concern about rodenticide introduction into bulk milk. Furthermore, the presence, detection, and characterization of rodenticides in milk have not been previously reported.

## 2. Materials and Methods

### 2.1. Rodenticides

Three rat poisons of analytical standard quality were used. Strychnine (Sigma-Aldrich, St. Louis, MO, USA) is a neurotoxin that induces seizures through binding to the neuron membranes and antagonizing glycine receptors in the central nervous system [19]. Bromadiolone (Sigma-Aldrich, St. Louis, MO, USA) and brodifacoum (Sigma-Aldrich, St. Louis, MO, USA) are both coumarin-derived anticoagulants that antagonize vitamin K1-epoxide [20], an enzyme critical for the proper coagulation of blood. It should be noted here that these three rodenticides are very toxic to humans and should, thus, be handled with extreme caution.

### 2.2. Determining LD50 Concentrations

The LD50, or median lethal dose, refers to the amount of a substance that is lethal to 50% of rats tested. We studied the effects of rodenticides introduced prior to coagulation, that is, present in the milk and calculated the LD50 concentrations of each of the toxins in the milk used in cheesemaking.

The acute LD50 of strychnine is 2.35 mg/kg b.w. (body weight) [21], that of bromadiolone is 1.5 mg/kg b.w. [22], and that of brodifacoum is 0.35 mg/kg b.w. [23]. To convert these to human LD50 levels, the average U.S. adult was found to be 80.5 kg [24] and average daily per capita U.S. consumption of cheese was determined to be 38.52 g [1], which amounts to about one or two slices of cheese per day. Assuming the rodenticide is evenly distributed throughout the milk (i.e., the ratio between rodenticide mass and dairy mass remains constant as the milk becomes cheese), we then calculated the toxin density in the milk. The density of the 3.25% fat milk (Harris Teeter, Matthews, NC, USA) used in the cheesemaking was calculated to be 1.013 g/mL [25]. These figures were then used to calculate the human LD50s of each select agent in milk. We determined that strychnine, bromadiolone, and brodifacoum have LD50 concentrations (mg/mL) in milk of 4.974, 3.175, and 0.741, respectively.

Minimum lethal dose (LDLo) data describes the lowest amount of toxin found to have caused a fatality in humans. For example, the strychnine LDLo’s for humans were 30 mg and 15 mg for an adult and child, respectively [26]. Thus, we calculated the minimum lethal concentration of strychnine as 0.39 mg/mL.

### 2.3. Expected Natural Variation

If rodenticide-induced changes in milk coagulation parameters are to be used as early warning signs of contamination, the changes must far exceed natural variation owing to routine changes in milk quality. Cassandro and colleagues investigated the variation in milk pH, gel strength, and RCT in a population of Holstein cows [9]. The group calculated the coefficient of variation (CV), or the standard deviation computed as a percentage of the mean, for each of the coagulation parameters. The CVs were reported as 2%, 35%, and 27% for pH, gel strength, and RCT, respectively. Since milk from the individual cows must be pooled together for bulk storage, we applied the sampling distribution. Intuitively, as more individual cows contribute milk to the storage tank, the less impact any individual daily variation will have on the final mixture. The average U.S. dairy farmstead claimed 120 dairy cows [27]. Thus, we expected bulk milk to comprise at least 120 samples from individual cows. By the sampling distribution, we calculated the CV for bulk milk pH as 2/sqrt (120) = 0.19%. Similarly, we calculated the bulk milk CVs for gel strength and RCT as 3.20% and 2.46%, respectively.

### 2.4. pH Analysis

Six 15-mL disposable tubes were used for each toxin. Grade A homogenized milk with a 3.25% fat content (Harris Teeter, Matthews, NC, USA) was stored at 4 °C. Ten milliliters of milk were added to each of the tubes. Due to the low aqueous solubility of the rodenticides, each one was dissolved in acetone at approximately 10 mg/mL acetone and added to the milk in three of the tubes at amounts that yielded toxin concentrations of 20%, 50%, and 80% of the toxin’s LD50—one level per tube. Acetone was added to the control tubes so that each pair of toxin and control tubes had equal total volumes. The six tubes were allowed to thermally equilibrate at 25 °C. The pH of each mixture was then calculated with an Accumet AR60 pH meter (Fisher Scientific, Waltham, MA, USA), and the values were recorded. The samples were again tested following overnight refrigeration at 4 °C. For each 25 °C sample, pH measurements were taken twice, and the entire procedure was performed in triplicate.

### 2.5. Rheological Measurements

The rennet used was Chy-Max Extra (Chr Hansen, Milwaukee, WI, USA) diluted 30 times in deionized water, prepared daily. Either the dissolved toxin, or acetone-only control, was added to the 3.25% fat milk. The mixture was then stirred (~10 s). Approximately 15 min later, the diluted rennet was added to the milk at a concentration of 2.4 μL rennet solution/mL milk, and a timer was started. The mixture was transferred into the serrated cup of StressTech Rheometer (Reologica Instruments, Bordentown, NJ, USA) until a thin layer covered the top of the serrated bob (approximately 13 mL). As the rheometer’s temperature control unit thermally equilibrated the sample at 31 °C over one minute, a layer of mineral oil was added to the top to prevent evaporation. At 3.5 min after rennet addition, the rotor lock was released, and preshear mixing began at 3 Hz for 0.5 min to achieve homogenous distribution of the toxin and rennet. This preshear mixing was followed by 0.5 min of final equilibrium time. For each rheological measurement, 0.5 Pa stress was applied to the sample, and the resultant strain and phase lag were recorded. Twenty measurements were taken at intervals of 3 min. The temperature was maintained at 31 °C (±0.1 °C). This test was first performed at 0.5 mg toxin/mL milk. If a difference from the control was noticed, that toxin was tested at lower concentrations. Tests on contaminated samples that demonstrated a difference in RCT or final gel strength from the control were duplicated.

### 2.6. Rotational Viscometer

A HAAKE Viscotester 7 plus (Thermo Scientific, Waltham, MA, USA) was used to monitor the changes in viscosity during milk coagulation. Five-hundred milliliters of the 3.25% fat milk were added to a clean 1000 mL beaker. Bromadiolone’s impact on milk coagulation was tested at 0, 2, and 4 mg in the 500 mL of milk in order to determine the effects of very low toxin concentrations. To achieve these bromadiolone concentrations, the toxin was first prepared in acetone at 10 mg toxin per 1 mL acetone and added to the milk. Control samples were prepared with both 200 and 400 μL acetone. The solutions were allowed to thermally equilibrate at 31 °C (±1 °C) for approximately 35 min in a water bath. Undiluted rennet was added at 0.08 μL rennet per 1 mL of milk, and a timer was started. The L1 spindle of the viscometer was lowered into the center of the sample and began rotating at 10 rpm. This procedure was performed in triplicate for the control samples and in duplicate for the contaminated samples.

### 2.7. Statistical Analysis

All statistical tests were performed with Excel software (Microsoft, Redmond, WA, USA). Comparisons were made using Student’s *t*-test and reported as two-tailed *p*-values.

## 3. Results and Discussion

### 3.1. Effects on pH

**Figure 1:**
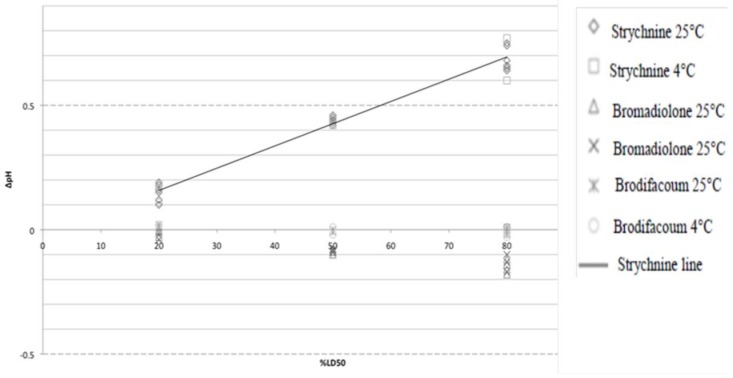
The effect of rodenticides on pH at 4 °C and 25 °C.

The value ∆pH was defined as the pH values of the control subtracted from the pH of the contaminated milk, each of which contained equal amounts of acetone. Strychnine and bromadiolone caused significant changes (*p* < 0.05) in pH at sub-LD50 levels, at both 4 °C and 25 °C. At sub-LD50 concentrations, both rodenticides produced changes in pH several times greater than that which would be expected due to natural variation. Brodifacoum yielded non-significant effects. The results are summarized in Figure 1: the effect of rodenticides on pH at 4 °C and 25 °C. The dashed horizontal lines at ±0.5 represent the detection threshold of the color-changing pH test papers with increments of one-half. The ∆pH values before and after overnight refrigeration were similar for all rodenticides.

While noticeable fluxes in pH may signal the possible presence of strychnine or bromadiolone, the former induces more substantial changes. This could be explained by the alkaline and acidic nature of the respective compounds. The buffering index (dB/dpH) of bovine milk decreases monotonically over the pH range from 5 to 8 [6]. Therefore, bovine milk exhibits a greater resistance to acidification than to alkalinization compared to the milk’s normal pH. We concluded that while pH could be used to detect sub-LD50 levels of certain toxins, it would be better utilized by helping to identify alkaline compounds. In addition, we noted that contamination with a strychnine salt instead of a free base may prevent this alarming pH change. Detection of bromadiolone poses a challenge due to its acidity, which can accelerate coagulation by decreasing milk pH [5]. This acceleration could, thus, counteract the observed rheological impacts of bromadiolone demonstrated in this study. Although brodifacoum is similarly acidic, its low LD50 (0.749 mg/mL) prevents significant pH changes at sub-LD50 concentrations.

### 3.2. Impact on Rheological Properties

**Figure 2:**
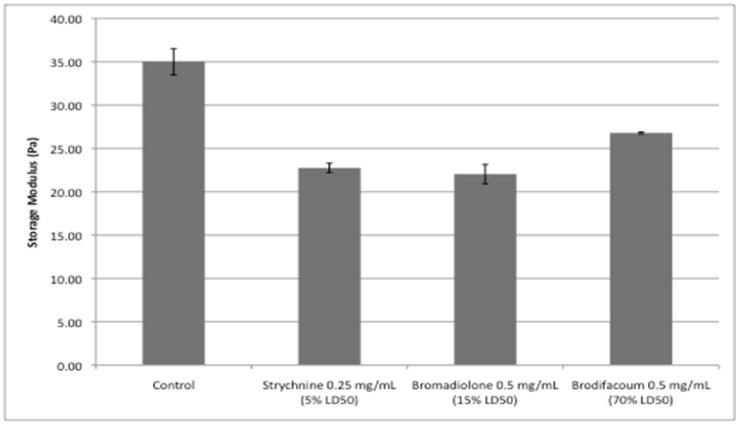
Impact of rodenticides on the coagulum strength of milk.

The storage modulus (G’), computed by the StressTech rheometer, was used to quantify he coagulum strength. The storage modulus represents elasticity and is calculated as follows: G’ = (τ/γ) × cos(φ), where τ is stress, γ is strain, and φ is phase lag. In this way, the storage modulus measures the sample’s resistance to displacement, and therefore correlates with gel strength. Comparing the storage moduli at 62 minutes since the rennet addition, Figure 2 demonstrates that sub-LD50 amounts of rodenticides in the milk significantly decreased the coagulum strength compared with the control (*p* < 0.05).

**Figure 3:**
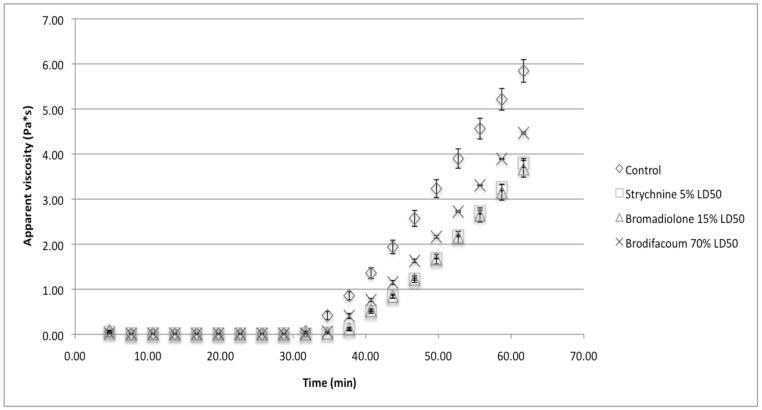
Impact of rodenticides on the apparent viscosity of milk.

The decreases were, on average, 26% of the control value, and all three exceeded seven times the expected bulk milk standard deviation of 3.20%, indicating that the changes would be drastic and noticeable. The StressTech rheometer also computed apparent viscosity (Pa·s). The linear rise in apparent viscosity observed in the rheometer for all samples was used to approximate the rennet coagulation time (RCT). A linear regression was performed on all viscosity data points greater than 0.01 Pa·s. By the method of Kopelman and Cogan [28], we used the x-intercept of this regression line in a viscosity vs. time plot (Figure 3) to approximate the RCT.

All three toxins significantly increased the RCT (*p* < 0.05) and strychnine and bromadiolone significantly reduced the slope of viscosity increase (*p* < 0.05). The control samples coagulated at approximately 30.5 min after rennet addition, and each rodenticide delayed coagulation by at least 4 min. Such delays correspond to more than five times the standard deviation of RCT expected from bulk milk.

The rotational viscometer data was also used to supplement the rheometer findings. The RCT was defined as the first increase from 0 Pa·s recorded by the viscometer. The addition of bromadiolone to the milk, increased the RCT noticeably but not significantly compared to the control. The other two toxins were only tested with the rheometer. The rotational viscometer could not be used to produce the same significant results as observed with the rheometer. This may be due to the breakage of the surrounding gel by the rotation of the probe as the gel starts to form. Therefore, the viscometer readings did not reflect the true nature of the milk gel. A vibrational system [8] would be more suitable for detecting the reduced gel strength of contaminated milk in a potential detection system. While contamination with strychnine sulfate instead of free base might avoid the pH effects, the significant effects on gel strength and RCT would remain. As little as 0.25 mg/mL strychnine substantially changed those rheological parameters. Consequently, by extrapolation, we estimated that this concentration of free base strychnine would increase milk pH by only 0.02. This increase in pH, taken alone, would only delay coagulation by 46 s, as approximated using the multivariate model of Daviau and colleagues [29]. The observed delay was 288 s. It is, thus, apparent that substitution with a strychnine salt would not avoid the noticeable impact on the rheological properties. In addition, it should be emphasized that the 0.25 mg/mL strychnine concentration falls below the 0.39 mg/mL concentration that corresponds to the lowest recorded lethal dose for a child. Thus, even truly sub-lethal doses of strychnine noticeably affect milk coagulation properties.

In the search for the mechanism of toxin interaction with the milk matrix, the similarities among the three toxins are illuminating. All three are hydrophobic and have high organic carbon adsorption coefficients (with K_oc_ > 1000). For example, one past study indicated that treatment with evaporated milk for strychnine poisoning increases its LD50 and reduces its concentration in the liver [30]. The milk appears to complex with strychnine, slowing absorption into the bloodstream. This past finding, as well as the high K_oc_ of the toxins, supports the hypothesis that the toxins adsorb onto the hydrophobic casein micelles. This adsorption could disrupt micelle-micelle interactions, thus reducing gel strength and delaying coagulation. Our findings are consistent with the previous observations as reported by Hathursinghe and Ibrahim [31], where authors reported that the presence of rodenticides, such as strychnine, bromadiolone, and brodifacoum, can negatively affect milk coagulation properties.

### 3.3. Limitations

One limitation of our work arises because the calculations presented in this paper assume a homogenous distribution of the contaminants throughout the dairy product as it separates from liquid milk into curd and whey. Since each of the toxins is hydrophobic, it may preferentially localize among the hydrophobic casein micelles of the curd compared to the aqueous whey. Therefore, the cheese could have a higher weight ratio of contaminants to dairy than the source milk. Further investigation is required to determine the exact relative concentrations, and to possibly adjust the LD50 calculations. An additional limitation is that we studied grocery milk in a laboratory setting. This ensured a more consistent comparison of control and experimental samples, but also excluded many of the variables intrinsic to a farmstead. We mitigated this problem by approximating the natural variations in bulk milk coagulation properties with real world data from cows and other sources of milk, such as buffalo, donkey, goat, sheep, and Jenny [32,33,34]. A logical next step would involve testing milk samples collected from bulk storage tanks in situ to validate the approximated bulk milk variations and the observed effects of rodenticide contamination.

## 4. Conclusions

This investigation demonstrated that sub-LD50 amounts of strychnine, bromadiolone, and brodifacoum significantly and noticeably alter the coagulation properties of milk, with applications to early detection of these contaminants in farm bulk storage tanks. The aberrations caused by the rodenticides lie more than five standard deviations outside the expected routine variations of bulk milk properties. While many factors can affect the coagulation properties studied in this paper, we quantitatively demonstrated that the rodenticides induced noticeable changes and, thus, warrant further consideration as preliminary warning signs of contamination. While aberrations may be caused by more innocuous chemicals, such as antibiotics, drastic changes nonetheless indicate that the milk is of dubious quality. Our investigation adds the novel contribution of rodenticides to the list of suspected causes.
